# First records of the Common Eastern Bumble Bee, *Bombus impatiens* Cresson (Hymenoptera: Apidae, Apinae, Bombini) from the Prairies Ecozone in Canada

**DOI:** 10.3897/BDJ.7.e30953

**Published:** 2019-01-10

**Authors:** Kirsten M. Palmier, Cory S. Sheffield

**Affiliations:** 1 Department of Biology, University of Regina, Regina, Canada Department of Biology, University of Regina Regina Canada; 2 Royal Saskatchewan Museum, Regina, Canada Royal Saskatchewan Museum Regina Canada

**Keywords:** non-native species, species at risk, pathogen spillover, Prairies, pollination, conservation, COSEWIC

## Abstract

**Background:**

In Canada, the Common Eastern Bumble Bee (*Bombus impatiens* Cresson) is native to southern Ontario and Quebec, but since being developed as a managed commercial pollinator, it has been exported to several other provinces for use in greenhouse and field crop settings. This has enabled this species to become established outside its natural range and it is now established in eastern Canada (New Brunswick, Nova Scotia, Prince Edward Island) and British Columbia. To date, the species has not been detected via field capture in the prairie provinces.

**New information:**

Here we report on recent captures of *B. impatiens* workers and males from south-eastern Alberta and suggest that these specimens escaped from nearby commercial greenhouses. The risk that the presence and looming establishment of this species has on native bumble bees in the Canadian prairies is discussed.

## Introduction

Non-native species pose one of the biggest threats to biodiversity due to their ability to outcompete native species for resources and the risks that they habour pathogens that can negatively impact local species (Wilson 1999, Gurevitch and Padilla 2004, Pimentel et al. 2005, Chivian and Bernstein 2008). Non-native species may establish by entirely natural phenomena or by unintentional means, such as through commerce (e.g. Sipes et al. 1996, Work et al. 2005). Other species are introduced on purpose for perceived benefits to humanity (e.g. Shine 2010, Aizen et al. 2018) though the potential impacts are not always thoroughly considered. The bees (Hymenoptera: Apoideae, Apiformes) illustrate several of these scenarios. Sheffield et al. 2011 reviewed the non-native bee species that were detected and/or or established in Canada up to that time, though other species have since been reported (Gibbs and Dathe 2017, Martins et al. 2017, Normandin et al. 2017). Of these, only the European Honey Bee (*Apis mellifera* L.) was intentionally introduced (Horn 2005), with the remaining species likely arriving incidentally in soil used as ship ballast (see Giles and Ascher 2006) or in plant stems (e.g. Ascher 2001, Gibbs and Sheffield 2009, Sheffield et al. 2011, Gibbs and Dathe 2017).

In recent decades, both real and perceived needs for supplemental crop pollination have led to the development, evaluation and/or management of non-*Apis* species for management. In Canada, this list includes the Alfalfa Leafcutter Bee, *Megachile rotundata* (Fabricius) (Megachilidae) (e.g. Javorek et al. 2002, Sheffield 2008), the Blue Orchard Bee, *Osmia lignaria* Say (Megachilidae) (e.g. Sheffield 2014, Sheffield et al. 2008a, Sheffield et al. 2008b, Frier et al. 2016b) and several bumble bee species, *Bombus* Latreille (Apidae) (Velthuis and van Doorn 2006, Strange 2015). For the latter, the most widely used is the Common Eastern Bumble Bee, *Bombus impatiens* Cresson (e.g. Whidden 1996, Stubbs and Drummond 2001, Velthuis and van Doorn 2006, Artz and Nault 2011, Campbell et al. 2017). Commercial use of bumble bee colonies began in Europe in 1987, where they were used to aid the pollination of tomato crops; use in Canada began in 1990 (Velthuis and van Doorn 2006). Commercially available bumble bees have been used to meet increasing global demaned for crop pollination (Velthuis and van Doorn 2006) where native pollinators are naturally low (Goulson et al. 2005, Ricketts et al. 2008) or have declined due to unfavourable agro-environmental effects (Batra 1995, Thorp 2003, Velthuis and van Doorn 2006).

*Bombus impatiens* is a native North American species and common in north-eastern North America (Mitchell 1962, Laverty and Harder 1988, Williams et al. 2014). In Canada, pre-1988, it had only been reported from Ontario (Mitchell 1962) and later the provinces of Ontario and Quebec, with one specimen known from north-western New Brunswick (Laverty and Harder 1988). Shortly after, this species became widely used as a commercial pollinator (Velthuis and van Doorn 2006) and by the early 1990s was used for blueberry pollination in New Brunswick (Whidden 1996); it was seemingly well established in Nova Scotia by the early 2000s (Sheffield et al. 2003). Its continued use for pollination in eastern Canada has led to its establishment (Williams et al. 2014) and its continued importation to other provinces, including British Columbia (Ratti and Colla 2010) and Newfoundland (Hicks 2011, Hicks and Sircom 2016) has led to its presence well outside its natural range. However, up to this point, *B. impatiens* has not been reported from the prairie provinces in Canada, though it had been used in controlled research projects in greenhouses (see Frier et al. 2016a). Our purpose here is to report on the first field-caught specimens of *B. impatiens* from the Canadian prairies and discuss the potential implications this has on native prairie bumble bees.

## Materials and methods

Field studies related to bumble bees in western Canada have been ongoing since 2013 (e.g. Sheffield et al. 2016), during which time many areas of western Canada were surveyed. Collection methods included netting bees from flowers to record floral usage and the use of Blue Vane Traps (Stephen and Rao 2007). Although many thousands of specimens were collected, below we report on and provide data for specimens of *B. impatiens* for this specific study. All specimens are deposited in the invertebrate zoology collection at the Royal Saskatchewan Museum (RSKM) and are being used for ongoing graduate research (by KP).

## Data resources

The full dataset for *Bombus impatiens* specimens that were used in this study is archived with Canadansys (http://community.canadensys.net/) under resource title "*Bombus impatiens* Cresson (Hymenoptera: Apidae, Apinae, Bombini) from the Prairies Ecozone in Canada" and can be accessed using the following: https://doi.org/10.5886/hugvqu. This resource has also been registered with GBIF, and assigned the following GBIF UUID: b065a152-9f83-4944-9ea0-545ac0703c35.

## Taxon treatments

### Bombus (Pyrobombus) impatiens

Cresson, 1863

Bombus impatiens Cresson 1863: 90 [♂]
Bombus (Pyrobombus) impatiens
Bombus (Pratobombus) impatiens var. deayi Chandler 1956: 116 [♀]
Bombus (Pyrobombus) impatiens

#### Materials

- **Type status:** Other material. **Occurrence:** catalogNumber: RSKM_ENT_E-181532; recordedBy: C. Sheffield, D. Fauser; individualCount: 1; sex: male; lifeStage: adult; **Taxon:** scientificName: Bombus impatiens; kingdom: Animalia; phylum: Arthropoda; class: Insecta; order: Hymenoptera; family: Apidae; genus: Bombus; subgenus: Pyrobombus; specificEpithet: impatiens; taxonRank: species; scientificNameAuthorship: Cresson, 1863; vernacularName: Common Eastern Bumble Bee; **Location:** country: Canada; stateProvince: Alberta; locality: 20 km south Medicine Hat; decimalLatitude: 49.9855; decimalLongitude: -110.75101; georeferenceProtocol: GPS; **Identification:** identifiedBy: Cory S. Sheffield; dateIdentified: 2018; **Event:** samplingProtocol: sweeping; year: 2014; month: 7; day: 1; habitat: roadside; Melilotus officinalis; **Record Level:** language: en; collectionID: urn:lsid:biocol.org:col:34252; institutionCode: RSKM; collectionCode: ENT; basisOfRecord: PreservedSpecimen
- **Type status:** Other material. **Occurrence:** catalogNumber: RSKM_ENT_E-181533; recordedBy: C. Sheffield, D. Fauser; individualCount: 1; sex: male; lifeStage: adult; **Taxon:** scientificName: Bombus impatiens; kingdom: Animalia; phylum: Arthropoda; class: Insecta; order: Hymenoptera; family: Apidae; genus: Bombus; subgenus: Pyrobombus; specificEpithet: impatiens; taxonRank: species; scientificNameAuthorship: Cresson, 1863; vernacularName: Common Eastern Bumble Bee; **Location:** country: Canada; stateProvince: Alberta; locality: 20 km south Medicine Hat; decimalLatitude: 49.9855; decimalLongitude: -110.75101; georeferenceProtocol: GPS; **Identification:** identifiedBy: Cory S. Sheffield; dateIdentified: 2018; **Event:** samplingProtocol: sweeping; year: 2014; month: 7; day: 1; habitat: roadside; Melilotus officinalis; **Record Level:** language: en; collectionID: urn:lsid:biocol.org:col:34252; institutionCode: RSKM; collectionCode: ENT; basisOfRecord: PreservedSpecimen
- **Type status:** Other material. **Occurrence:** catalogNumber: RSKM_ENT_E-181534; recordedBy: C. Sheffield, D. Fauser; individualCount: 1; sex: male; lifeStage: adult; **Taxon:** scientificName: Bombus impatiens; kingdom: Animalia; phylum: Arthropoda; class: Insecta; order: Hymenoptera; family: Apidae; genus: Bombus; subgenus: Pyrobombus; specificEpithet: impatiens; taxonRank: species; scientificNameAuthorship: Cresson, 1863; vernacularName: Common Eastern Bumble Bee; **Location:** country: Canada; stateProvince: Alberta; locality: 20 km south Medicine Hat; decimalLatitude: 49.9855; decimalLongitude: -110.75101; georeferenceProtocol: GPS; **Identification:** identifiedBy: Cory S. Sheffield; dateIdentified: 2018; **Event:** samplingProtocol: sweeping; year: 2014; month: 7; day: 1; habitat: roadside; Melilotus officinalis; **Record Level:** language: en; collectionID: urn:lsid:biocol.org:col:34252; institutionCode: RSKM; collectionCode: ENT; basisOfRecord: PreservedSpecimen
- **Type status:** Other material. **Occurrence:** catalogNumber: RSKM_ENT_E-181535; recordedBy: C. Sheffield, D. Fauser; individualCount: 1; sex: female; lifeStage: adult; **Taxon:** scientificName: Bombus impatiens; kingdom: Animalia; phylum: Arthropoda; class: Insecta; order: Hymenoptera; family: Apidae; genus: Bombus; subgenus: Pyrobombus; specificEpithet: impatiens; taxonRank: species; scientificNameAuthorship: Cresson, 1863; vernacularName: Common Eastern Bumble Bee; **Location:** country: Canada; stateProvince: Alberta; locality: 20 km south Medicine Hat; decimalLatitude: 49.9855; decimalLongitude: -110.75101; georeferenceProtocol: GPS; **Identification:** identifiedBy: Cory S. Sheffield; dateIdentified: 2018; **Event:** samplingProtocol: sweeping; year: 2014; month: 7; day: 1; habitat: roadside; Melilotus officinalis; **Record Level:** language: en; collectionID: urn:lsid:biocol.org:col:34252; institutionCode: RSKM; collectionCode: ENT; basisOfRecord: PreservedSpecimen
- **Type status:** Other material. **Occurrence:** catalogNumber: RSKM_ENT_E-173057; recordedBy: C. Sheffield, D. Fauser; individualCount: 1; sex: female; lifeStage: adult; **Taxon:** scientificName: Bombus impatiens; kingdom: Animalia; phylum: Arthropoda; class: Insecta; order: Hymenoptera; family: Apidae; genus: Bombus; specificEpithet: impatiens; scientificNameAuthorship: Cresson, 1863; vernacularName: Common Eastern Bumble Bee; **Location:** continent: North America; country: Canada; stateProvince: Alberta; locality: Redcliff; decimalLatitude: 50.07984; decimalLongitude: -110.77859; **Identification:** identifiedBy: C. Sheffield; dateIdentified: 2018; **Event:** year: 2016; month: 8; day: 10; **Record Level:** collectionCode: Insects; ownerInstitutionCode: RSKM; basisOfRecord: PreservedSpecimen
- **Type status:** Other material. **Occurrence:** catalogNumber: RSKM_ENT_E-173058; recordedBy: C. Sheffield, D. Fauser; individualCount: 1; sex: male; lifeStage: adult; **Taxon:** scientificName: Bombus impatiens; kingdom: Animalia; phylum: Arthropoda; class: Insecta; order: Hymenoptera; family: Apidae; genus: Bombus; specificEpithet: impatiens; scientificNameAuthorship: Cresson, 1863; vernacularName: Common Eastern Bumble Bee; **Location:** continent: North America; country: Canada; stateProvince: Alberta; locality: Redcliff; decimalLatitude: 50.07984; decimalLongitude: -110.77859; **Identification:** identifiedBy: C. Sheffield; dateIdentified: 2018; **Event:** year: 2016; month: 8; day: 10; **Record Level:** collectionCode: Insects; ownerInstitutionCode: RSKM; basisOfRecord: PreservedSpecimen
- **Type status:** Other material. **Occurrence:** catalogNumber: RSKM_ENT_E-173059; recordedBy: C. Sheffield, D. Fauser; individualCount: 1; sex: female; lifeStage: adult; **Taxon:** scientificName: Bombus impatiens; kingdom: Animalia; phylum: Arthropoda; class: Insecta; order: Hymenoptera; family: Apidae; genus: Bombus; specificEpithet: impatiens; scientificNameAuthorship: Cresson, 1863; vernacularName: Common Eastern Bumble Bee; **Location:** continent: North America; country: Canada; stateProvince: Alberta; locality: Redcliff; decimalLatitude: 50.07984; decimalLongitude: -110.77859; **Identification:** identifiedBy: C. Sheffield; dateIdentified: 2018; **Event:** year: 2016; month: 8; day: 10; **Record Level:** collectionCode: Insects; ownerInstitutionCode: RSKM; basisOfRecord: PreservedSpecimen
- **Type status:** Other material. **Occurrence:** catalogNumber: RSKM_ENT_E-173060; recordedBy: C. Sheffield, D. Fauser; individualCount: 1; sex: male; lifeStage: adult; **Taxon:** scientificName: Bombus impatiens; kingdom: Animalia; phylum: Arthropoda; class: Insecta; order: Hymenoptera; family: Apidae; genus: Bombus; specificEpithet: impatiens; scientificNameAuthorship: Cresson, 1863; vernacularName: Common Eastern Bumble Bee; **Location:** continent: North America; country: Canada; stateProvince: Alberta; locality: Redcliff; decimalLatitude: 50.07984; decimalLongitude: -110.77859; **Identification:** identifiedBy: C. Sheffield; dateIdentified: 2018; **Event:** year: 2016; month: 8; day: 10; **Record Level:** collectionCode: Insects; ownerInstitutionCode: RSKM; basisOfRecord: PreservedSpecimen
- **Type status:** Other material. **Occurrence:** catalogNumber: RSKM_ENT_E-173061; recordedBy: C. Sheffield, D. Fauser; individualCount: 1; sex: male; lifeStage: adult; **Taxon:** scientificName: Bombus impatiens; kingdom: Animalia; phylum: Arthropoda; class: Insecta; order: Hymenoptera; family: Apidae; genus: Bombus; specificEpithet: impatiens; scientificNameAuthorship: Cresson, 1863; vernacularName: Common Eastern Bumble Bee; **Location:** continent: North America; country: Canada; stateProvince: Alberta; locality: Redcliff; decimalLatitude: 50.07984; decimalLongitude: -110.77859; **Identification:** identifiedBy: C. Sheffield; dateIdentified: 2018; **Event:** year: 2016; month: 8; day: 10; **Record Level:** collectionCode: Insects; ownerInstitutionCode: RSKM; basisOfRecord: PreservedSpecimen
- **Type status:** Other material. **Occurrence:** catalogNumber: RSKM_ENT_E-173062; recordedBy: C. Sheffield, D. Fauser; individualCount: 1; sex: female; lifeStage: adult; **Taxon:** scientificName: Bombus impatiens; kingdom: Animalia; phylum: Arthropoda; class: Insecta; order: Hymenoptera; family: Apidae; genus: Bombus; specificEpithet: impatiens; scientificNameAuthorship: Cresson, 1863; vernacularName: Common Eastern Bumble Bee; **Location:** continent: North America; country: Canada; stateProvince: Alberta; locality: Redcliff; decimalLatitude: 50.07984; decimalLongitude: -110.77859; **Identification:** identifiedBy: C. Sheffield; dateIdentified: 2018; **Event:** year: 2016; month: 8; day: 10; **Record Level:** collectionCode: Insects; ownerInstitutionCode: RSKM; basisOfRecord: PreservedSpecimen
- **Type status:** Other material. **Occurrence:** catalogNumber: RSKM_ENT_E-173063; recordedBy: C. Sheffield, D. Fauser; individualCount: 1; sex: female; lifeStage: adult; **Taxon:** scientificName: Bombus impatiens; kingdom: Animalia; phylum: Arthropoda; class: Insecta; order: Hymenoptera; family: Apidae; genus: Bombus; specificEpithet: impatiens; scientificNameAuthorship: Cresson, 1863; vernacularName: Common Eastern Bumble Bee; **Location:** continent: North America; country: Canada; stateProvince: Alberta; locality: Redcliff; decimalLatitude: 50.07984; decimalLongitude: -110.77859; **Identification:** identifiedBy: C. Sheffield; dateIdentified: 2018; **Event:** year: 2016; month: 8; day: 10; **Record Level:** collectionCode: Insects; ownerInstitutionCode: RSKM; basisOfRecord: PreservedSpecimen
- **Type status:** Other material. **Occurrence:** catalogNumber: RSKM_ENT_E-189525; recordedBy: K. Palmier; individualCount: 1; sex: male; lifeStage: adult; **Taxon:** scientificName: Bombus impatiens; kingdom: Animalia; phylum: Arthropoda; class: Insecta; order: Hymenoptera; family: Apidae; genus: Bombus; specificEpithet: impatiens; scientificNameAuthorship: Cresson, 1863; vernacularName: Common Eastern Bumble Bee; **Location:** continent: North America; country: Canada; stateProvince: Alberta; locality: Redcliff; decimalLatitude: 50.0752; decimalLongitude: -110.7905; **Identification:** identifiedBy: K. Palmier; dateIdentified: 2018; **Event:** samplingProtocol: Blue Vane Trap; verbatimEventDate: 22-Jun-2018 to 13-Jul-2018; **Record Level:** collectionCode: Insects; ownerInstitutionCode: RSKM; basisOfRecord: PreservedSpecimen
- **Type status:** Other material. **Occurrence:** catalogNumber: RSKM_ENT_E-189530; recordedBy: K. Palmier; individualCount: 1; sex: female; lifeStage: adult; **Taxon:** scientificName: Bombus impatiens; kingdom: Animalia; phylum: Arthropoda; class: Insecta; order: Hymenoptera; family: Apidae; genus: Bombus; specificEpithet: impatiens; scientificNameAuthorship: Cresson, 1863; vernacularName: Common Eastern Bumble Bee; **Location:** continent: North America; country: Canada; stateProvince: Alberta; locality: Redcliff; decimalLatitude: 50.0752; decimalLongitude: -110.7905; **Identification:** identifiedBy: K. Palmier; dateIdentified: 2018; **Event:** samplingProtocol: Blue Vane Trap; verbatimEventDate: 22-Jun-2018 to 13-Jul-2018; **Record Level:** collectionCode: Insects; ownerInstitutionCode: RSKM; basisOfRecord: PreservedSpecimen
- **Type status:** Other material. **Occurrence:** catalogNumber: RSKM_ENT_E-189529; recordedBy: K. Palmier; individualCount: 1; sex: female; lifeStage: adult; **Taxon:** scientificName: Bombus impatiens; kingdom: Animalia; phylum: Arthropoda; class: Insecta; order: Hymenoptera; family: Apidae; genus: Bombus; specificEpithet: impatiens; scientificNameAuthorship: Cresson, 1863; vernacularName: Common Eastern Bumble Bee; **Location:** continent: North America; country: Canada; stateProvince: Alberta; locality: Redcliff; decimalLatitude: 50.0752; decimalLongitude: -110.7905; **Identification:** identifiedBy: K. Palmier; dateIdentified: 2018; **Event:** samplingProtocol: Blue Vane Trap; verbatimEventDate: 22-Jun-2018 to 13-Jul-2018; **Record Level:** collectionCode: Insects; ownerInstitutionCode: RSKM; basisOfRecord: PreservedSpecimen
- **Type status:** Other material. **Occurrence:** catalogNumber: RSKM_ENT_E-189528; recordedBy: K. Palmier; individualCount: 1; sex: female; lifeStage: adult; **Taxon:** scientificName: Bombus impatiens; kingdom: Animalia; phylum: Arthropoda; class: Insecta; order: Hymenoptera; family: Apidae; genus: Bombus; specificEpithet: impatiens; scientificNameAuthorship: Cresson, 1863; vernacularName: Common Eastern Bumble Bee; **Location:** continent: North America; country: Canada; stateProvince: Alberta; locality: Redcliff; decimalLatitude: 50.0752; decimalLongitude: -110.7905; **Identification:** identifiedBy: K. Palmier; dateIdentified: 2018; **Event:** samplingProtocol: Blue Vane Trap; verbatimEventDate: 22-Jun-2018 to 13-Jul-2018; **Record Level:** collectionCode: Insects; ownerInstitutionCode: RSKM; basisOfRecord: PreservedSpecimen
- **Type status:** Other material. **Occurrence:** catalogNumber: RSKM_ENT_E-189527; recordedBy: K. Palmier; individualCount: 1; sex: female; lifeStage: adult; **Taxon:** scientificName: Bombus impatiens; kingdom: Animalia; phylum: Arthropoda; class: Insecta; order: Hymenoptera; family: Apidae; genus: Bombus; specificEpithet: impatiens; scientificNameAuthorship: Cresson, 1863; vernacularName: Common Eastern Bumble Bee; **Location:** continent: North America; country: Canada; stateProvince: Alberta; locality: Redcliff; decimalLatitude: 50.0752; decimalLongitude: -110.7905; **Identification:** identifiedBy: K. Palmier; dateIdentified: 2018; **Event:** samplingProtocol: Blue Vane Trap; verbatimEventDate: 22-Jun-2018 to 13-Jul-2018; **Record Level:** collectionCode: Insects; ownerInstitutionCode: RSKM; basisOfRecord: PreservedSpecimen
- **Type status:** Other material. **Occurrence:** catalogNumber: RSKM_ENT_E-189526; recordedBy: K. Palmier; individualCount: 1; sex: female; lifeStage: adult; **Taxon:** scientificName: Bombus impatiens; kingdom: Animalia; phylum: Arthropoda; class: Insecta; order: Hymenoptera; family: Apidae; genus: Bombus; specificEpithet: impatiens; scientificNameAuthorship: Cresson, 1863; vernacularName: Common Eastern Bumble Bee; **Location:** continent: North America; country: Canada; stateProvince: Alberta; locality: Redcliff; decimalLatitude: 50.0752; decimalLongitude: -110.7905; **Identification:** identifiedBy: K. Palmier; dateIdentified: 2018; **Event:** samplingProtocol: Blue Vane Trap; verbatimEventDate: 22-Jun-2018 to 13-Jul-2018; **Record Level:** collectionCode: Insects; ownerInstitutionCode: RSKM; basisOfRecord: PreservedSpecimen
- **Type status:** Other material. **Occurrence:** catalogNumber: RSKM_ENT_E-197326; recordedBy: K. Palmier; individualCount: 1; sex: female; lifeStage: adult; **Taxon:** scientificName: Bombus impatiens; kingdom: Animalia; phylum: Arthropoda; class: Insecta; order: Hymenoptera; family: Apidae; genus: Bombus; specificEpithet: impatiens; scientificNameAuthorship: Cresson, 1863; vernacularName: Common Eastern Bumble Bee; **Location:** continent: North America; country: Canada; stateProvince: Alberta; locality: Redcliff; decimalLatitude: 50.0752; decimalLongitude: -110.7079; **Identification:** identifiedBy: K. Palmier; dateIdentified: 2018; **Event:** samplingProtocol: Blue Vane Trap; verbatimEventDate: 13-Jun-2018 to 13-Jul-2018; **Record Level:** collectionCode: Insects; ownerInstitutionCode: RSKM; basisOfRecord: PreservedSpecimen
- **Type status:** Other material. **Occurrence:** catalogNumber: RSKM_ENT_E-197327; recordedBy: K. Palmier; individualCount: 1; sex: female; lifeStage: adult; **Taxon:** scientificName: Bombus impatiens; kingdom: Animalia; phylum: Arthropoda; class: Insecta; order: Hymenoptera; family: Apidae; genus: Bombus; specificEpithet: impatiens; scientificNameAuthorship: Cresson, 1863; vernacularName: Common Eastern Bumble Bee; **Location:** continent: North America; country: Canada; stateProvince: Alberta; locality: Redcliff; decimalLatitude: 50.0752; decimalLongitude: -110.7079; **Identification:** identifiedBy: K. Palmier; dateIdentified: 2018; **Event:** samplingProtocol: Blue Vane Trap; verbatimEventDate: 13-Jun-2018 to 13-Jul-2018; **Record Level:** collectionCode: Insects; ownerInstitutionCode: RSKM; basisOfRecord: PreservedSpecimen
- **Type status:** Other material. **Occurrence:** catalogNumber: RSKM_ENT_E-197484; recordedBy: K. Palmier; individualCount: 1; sex: male; lifeStage: adult; **Taxon:** scientificName: Bombus impatiens; kingdom: Animalia; phylum: Arthropoda; class: Insecta; order: Hymenoptera; family: Apidae; genus: Bombus; specificEpithet: impatiens; scientificNameAuthorship: Cresson, 1863; vernacularName: Common Eastern Bumble Bee; **Location:** continent: North America; country: Canada; stateProvince: Alberta; locality: Redcliff; decimalLatitude: 50.0657; decimalLongitude: -110.778; **Identification:** identifiedBy: K. Palmier; dateIdentified: 2018; **Event:** samplingProtocol: Blue Vane Trap; verbatimEventDate: 13-Jun-2018 to 13-Jul-2018; **Record Level:** collectionCode: Insects; ownerInstitutionCode: RSKM; basisOfRecord: PreservedSpecimen
- **Type status:** Other material. **Occurrence:** catalogNumber: RSKM_ENT_E-197486; recordedBy: K. Palmier; individualCount: 1; sex: female; lifeStage: adult; **Taxon:** scientificName: Bombus impatiens; kingdom: Animalia; phylum: Arthropoda; class: Insecta; order: Hymenoptera; family: Apidae; genus: Bombus; specificEpithet: impatiens; scientificNameAuthorship: Cresson, 1863; vernacularName: Common Eastern Bumble Bee; **Location:** continent: North America; country: Canada; stateProvince: Alberta; locality: Redcliff; decimalLatitude: 50.0657; decimalLongitude: -110.778; **Identification:** identifiedBy: K. Palmier; dateIdentified: 2018; **Event:** samplingProtocol: Blue Vane Trap; verbatimEventDate: 13-Jun-2018 to 13-Jul-2018; **Record Level:** collectionCode: Insects; ownerInstitutionCode: RSKM; basisOfRecord: PreservedSpecimen
- **Type status:** Other material. **Occurrence:** catalogNumber: RSKM_ENT_E-197485; recordedBy: K. Palmier; individualCount: 1; sex: female; lifeStage: adult; **Taxon:** scientificName: Bombus impatiens; kingdom: Animalia; phylum: Arthropoda; class: Insecta; order: Hymenoptera; family: Apidae; genus: Bombus; specificEpithet: impatiens; scientificNameAuthorship: Cresson, 1863; vernacularName: Common Eastern Bumble Bee; **Location:** continent: North America; country: Canada; stateProvince: Alberta; locality: Redcliff; decimalLatitude: 50.0657; decimalLongitude: -110.778; **Identification:** identifiedBy: K. Palmier; dateIdentified: 2018; **Event:** samplingProtocol: Blue Vane Trap; verbatimEventDate: 13-Jun-2018 to 13-Jul-2018; **Record Level:** collectionCode: Insects; ownerInstitutionCode: RSKM; basisOfRecord: PreservedSpecimen
- **Type status:** Other material. **Occurrence:** catalogNumber: RSKM_ENT_E-197522; recordedBy: K. Palmier; individualCount: 1; sex: female; lifeStage: adult; **Taxon:** scientificName: Bombus impatiens; kingdom: Animalia; phylum: Arthropoda; class: Insecta; order: Hymenoptera; family: Apidae; genus: Bombus; specificEpithet: impatiens; scientificNameAuthorship: Cresson, 1863; vernacularName: Common Eastern Bumble Bee; **Location:** continent: North America; country: Canada; stateProvince: Alberta; locality: Redcliff; decimalLatitude: 50.083; decimalLongitude: -110.8163; **Identification:** identifiedBy: K. Palmier; dateIdentified: 2018; **Event:** samplingProtocol: Blue Vane Trap; verbatimEventDate: 13-Jun-2018 to 13-Jul-2018; **Record Level:** collectionCode: Insects; ownerInstitutionCode: RSKM; basisOfRecord: PreservedSpecimen
- **Type status:** Other material. **Occurrence:** catalogNumber: RSKM_ENT_E-197523; recordedBy: K. Palmier; individualCount: 1; sex: female; lifeStage: adult; **Taxon:** scientificName: Bombus impatiens; kingdom: Animalia; phylum: Arthropoda; class: Insecta; order: Hymenoptera; family: Apidae; genus: Bombus; specificEpithet: impatiens; scientificNameAuthorship: Cresson, 1863; vernacularName: Common Eastern Bumble Bee; **Location:** continent: North America; country: Canada; stateProvince: Alberta; locality: Redcliff; decimalLatitude: 50.083; decimalLongitude: -110.8163; **Identification:** identifiedBy: K. Palmier; dateIdentified: 2018; **Event:** samplingProtocol: Blue Vane Trap; verbatimEventDate: 13-Jun-2018 to 13-Jul-2018; **Record Level:** collectionCode: Insects; ownerInstitutionCode: RSKM; basisOfRecord: PreservedSpecimen

#### Diagnosis

**Figure 1.**
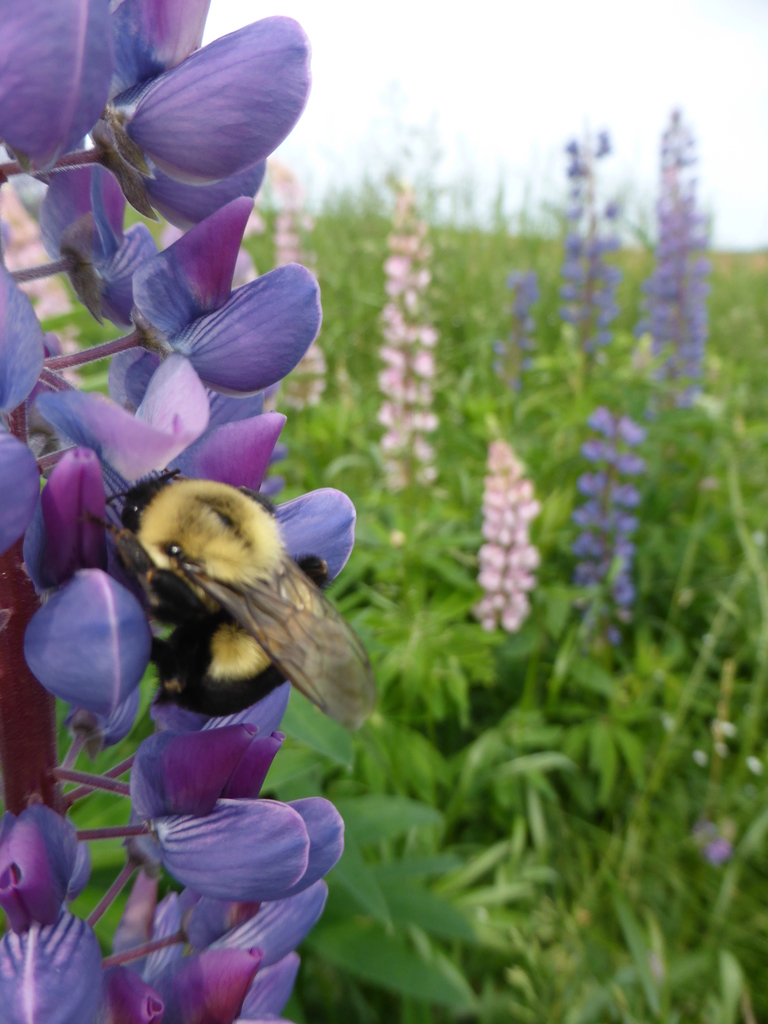
Female *Bombus impatiens* Cresson foraging on *Lupinus polyphyllus* Lindl. near Souris, Prince Edward Island, Canada, where the species is not native, but has established. This specimen shows the typical colouration for the species. Photo by Cory S. Sheffield.

*Bombus impatiens* is morphologically unique amongst other North American bumble bees in that both males and females have only the first metasomal tergum with pale pubescence, the remaining terga are entirely black (Fig. 1). On rare instances, some individuals may have the second and third terga with some orange pubescence medially, hence the form described by Chandler (1956) (see Taxon Treatment above).

#### Distribution

In Canada, this species native range includes southern Ontario, Quebec and perhaps adjacent New Brunswick (Laverty and Harder 1988), but has established throughout the Maritime Provinces (Sheffield et al. 2003, Williams et al. 2014) and south-western British Columbia (Ratti and Colla 2010) and has been used commercially in field settings in Newfoundland (Hicks 2011, Hicks and Sircom 2016, Hicks et al. 2018). In the United States, it is widespread in the east, with only a handful of records west of Texas (Williams et al. 2014).

## Discussion

The documentation of worker and male *Bombus impatiens* within south-eastern Alberta (Figs 2, 4) confirms that colonies are used for the pollination of greenhouse crops. Redcliff, Alberta has one of the largest greenhouse operations in western Canada (Laate 2013) and recent capture of specimens of *B. impatiens* in surveys in the Canadian prairies suggests that it is also possible that mated queens may have escaped from greenhouses, allowing this species to establish. Federal government guidelines for the use of commercial colonies of *B. impatiens* in Canada recommend the use of queen excluders when used outside of its natural range (Canadian Food Inspection Agency 2013), which would greatly reduce the probability of this happening. However, this practice does not necessarily lower the impact that the presence of this species may have on native bumble bees and presumably this practice has not been used or been successful in Atlantic Canada where the species has established (Fig. 1). Previous studies (Colla et al. 2006, Otterstatter and Thomson 2008) in Ontario, Canada and elsewhere (Sachman-Ruiz et al. 2015) discuss increased pathogen loads in native bumble bees collected adjacent to greenhouses that were using commercial bumble bee colonies. In addition, Hicks et al. (2018) recently reported that native bumble bees will enter commercial colonies and are likely to pick up pathogens. As such, the use of commercially managed bumble bees in open systems may have severe impacts on native species, even without the species establishing (Kelly et al. 2009, Graystock et al. 2015).

**Figure 3.**
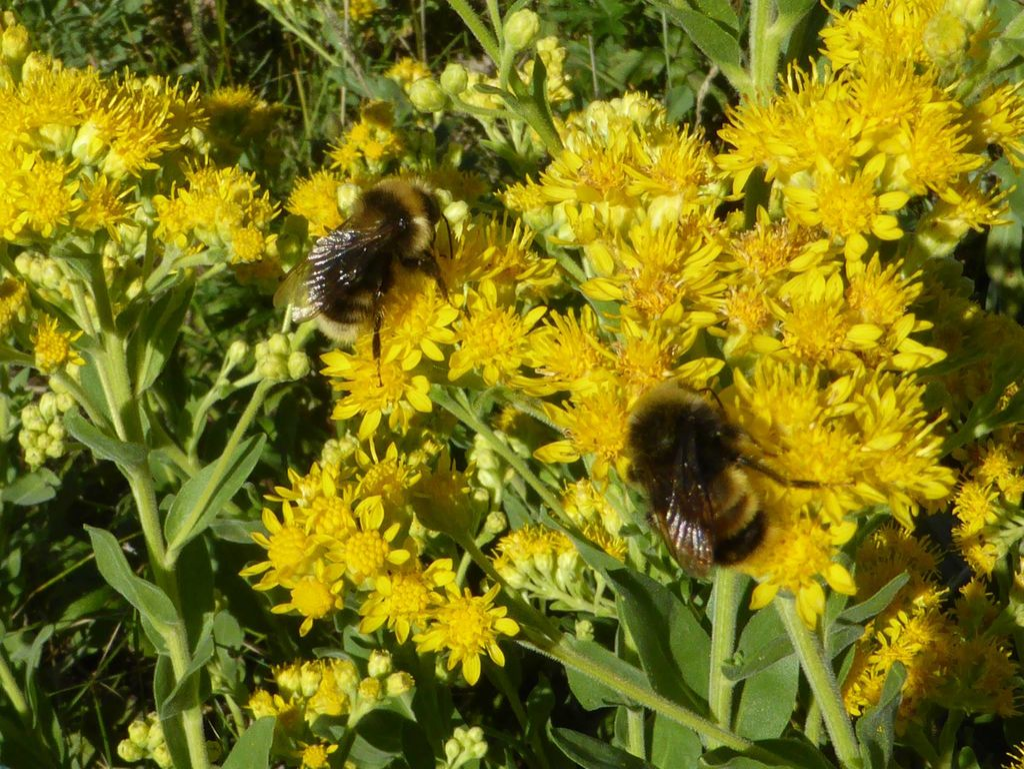
Male *Bombus occidentalis* Greene (background) and *B. terricola* Kirby (foreground) in Regina, Saskatchewan, possibly the most eastern location in Canada where both species at risk co-occur naturally. Photo by Cory S. Sheffield.

**Figure 4.**
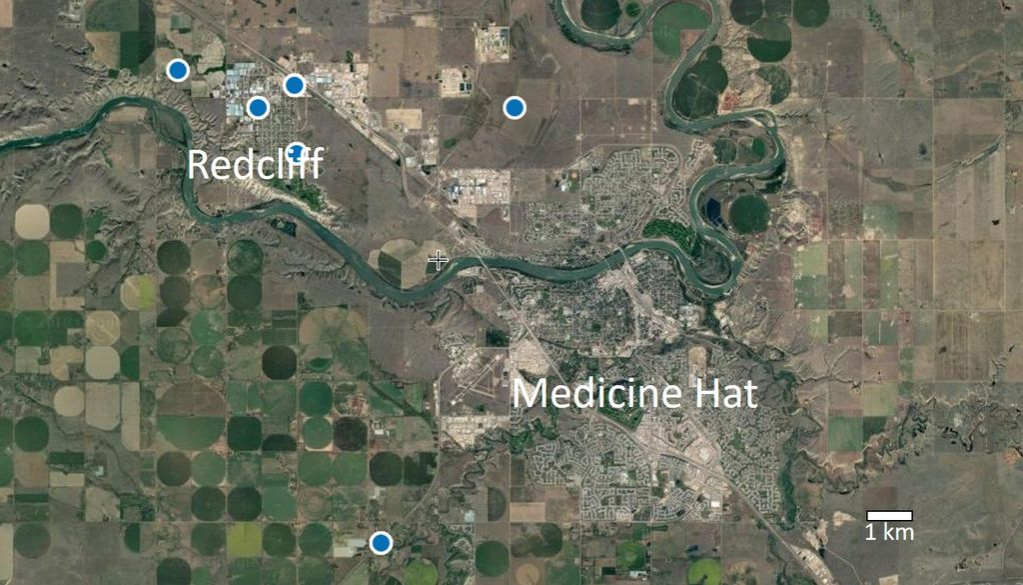
Distribution map of *Bombus impatiens* Cresson (Hymenoptera: Apidae) captures in south-eastern Alberta, Canada from 2015-2018.

Several bumble bee species have declined in abundance in North America (Grixti et al. 2009), including Canada (Colla and Packer 2008, Colla et al. 2012, Sheffield et al. 2016). National conservation status assessments by the Committee on the Status of Endangered Wildlife in Canada (COSEWIC) for *Bombus occidentalis* Greene (COSEWIC 2014a) and its sister species, *B. terricola* (COSEWIC 2015), have indicated that declines for these species have been mostly limited to southern British Columbia and eastern Canada, respectively, with declines not supported in most of the Canadian prairies where both species co-occur as far east as Saskatchewan (Fig. 3). Although the Prairies Ecozone is not considered the historic stronghold for either of these species in Canada (see Williams et al. 2014), they have been present in low but stable numbers (COSEWIC 2014a, COSEWIC 2015, Sheffield et al. 2016). Furthermore, the declines of these species and other members of the subgenus Bombus (COSEWIC 2010) have had a cascading effects on the cuckoo bumble bees (subgenus Psithyrus) that use them as hosts, with one once widespread species, *B. bohemicus* now only found in largely undisturbed areas in the northwest of Canada (COSEWIC 2014b).

As a result of these declines, researchers have been exploring the emergence and spread of infectious diseases during interactions between commercially managed species and local wild populations (Colla et al. 2006, Colla and Ratti 2010, Cameron et al. 2011, Colla et al. 2012, Evison et al. 2012, Graystock et al. 2013, Graystock et al. 2014, Graystock et al. 2015, Graystock et al. 2016). When managed species are allowed to co-exist with wild populations, physical contact between infected and non-infected individuals can result in direct transmission of pathogens or pathogens can be transmitted indirectly by vectors (Graystock et al. 2015). Managed bees can be/are transported over large distances and can introduce new and unusual pathogens to native wild populations (Otterstatter and Thomson 2008, Goulson 2010, Graystock et al. 2013, Goulson and Hughes 2015, Aizen et al. 2018, Hicks et al. 2018). Pathogen spillover occurs when a reservoir population, such as commercial managed bees, transmit pathogens to wild populations (Power and Mitchell 2004). Furthermore, the large size and density of managed populations makes them favourable for the emergence, evolution and propagation of pathogens that demonstrate improved transmissibility. Multiple studies have suggested that pathogen spillover in North America is linked to bumble bee population declines (Colla et al. 2006, Otterstatter and Thomson 2008, Szabo et al. 2012).

There are many complicated relationships between pathogens and their hosts and the severity of disease depends on the host health and the virulence of the pathogen. All animals, including insects, are more susceptible to disease when stressed by adverse ecological pressures (Jokela et al. 2005). Thus, when introduced species cause stress by competing with native species (Ings et al. 2006), the increased stress results in susceptibility to higher pathogen loads and increased severity of disease (Graystock et al. 2016). Disease outbreaks can exacerbate challenges in already struggling populations, pushing them closer to extinction (Jokela et al. 2005). The potential establishment of *B. impatiens* in south-eastern Alberta thus presents a great risk to native prairie bumble bees, particularly as native bumble bee species have experienced acute declines in other parts of Canada (Colla and Packer 2008). Nevertheless, managed bees that have escaped from greenhouses do not need to become established for pathogen spillover or competition of resources to occur.

The presence and looming establishment of *B. impatiens* in south-eastern Alberta thus presents a great risk to native prairie bumble bees, particularly the three nationally assessed species that have undergone declines in other parts of Canada. Recently, Kent et al. (2018) reported on the conservation genomics of *B. terricola* populations in Ontario and Quebec. According to that study, *B. terricola* likely underwent a severe population crash after the last Ice Age, resulting in small, inbred populations (Kent et al. 2018). Although this species subsequently expanded and became established throughout much of Canada and the United States (Williams et al. 2014), rapid declines have been observed in recent decades (Grixti et al. 2009, Cameron et al. 2011, Colla et al. 2012, Sheffield et al. 2016). The bottleneck in *B. terricola*'s diversity may have left this species susceptible to new pathogens, at least in eastern North America (Kent et al. 2018). Although *B. terricola* was assessed as “Threatened” by the International Union for the Conservation of Nature and severe declines have occurred in eastern and central Canada, these declines have been offset by stable populations in other parts of Canada, including the prairies, resulting in its assessment at the lower conservation priority “Special Concern” by COSEWIC (COSEWIC 2015). Eastern Canadian populations demonstrate immune-related gene signatures, implicating infectious disease pressures (Kent et al. 2018), but it is unknown if western populations exhibit the same signatures. If *B. terricola* is declining due to pathogen pressures, this raises concerns for closely-related North American bumble bees in the same subgenus, *Bombus* (Hines 2008). Four out of five of these species are already recognised as species at risk (three of these assessed in Canada), yet it remains unclear how any of these other bee species, including *B. occidentalis* and *B. cryptarum*, will respond to further establishment of *B. impatiens* in Alberta.

## Supplementary Material

XML Treatment for Bombus (Pyrobombus) impatiens

## Figures and Tables

**Figure 2a.**
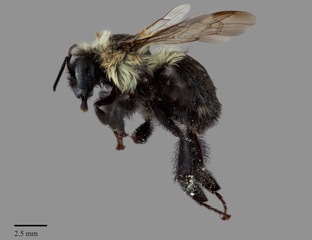
female worker

**Figure 2b.**
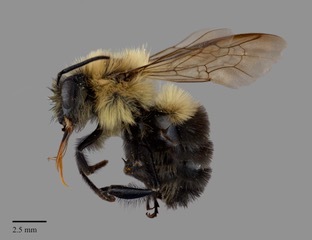
male

## References

[B4736457] Aizen M. A., Smith-Ramírez C., Morales C. L., Vieli L., Sáez A., Barahona-Segovia R. M., Arbetman M. P., Montalva J., Garibaldi L. A., Inouye D. W., Harder L. D. (2018). Coordinated species importation policies are needed to reduce serious invasions globally: the case of alien bumblebees in South America. Journal of Applied Ecology.

[B4736474] Artz D. R., Nault B. A. (2011). Performance of *Apis
mellifera*, *Bombus
impatiens*, and *Peponapis
pruinosa* (Hymenoptera: Apidae) as Pollinators of Pumpkin. Journal of Economic Entomology.

[B4720473] Ascher J. S. (2001). *Hylaeus
hyalinatus* Smith, a European bee new to North America, with notes on other adventive bees (Hymenoptera: Apoidea). Proceedings of the Entomological Society of Washington.

[B4736252] Batra S. W. T. (1995). Bees and pollination in our changing environment. Apidologie.

[B4699346] Cameron S. A., Lozier J. D., Strange J. P., Koch J. B., Cordes N., Solter L. F., Griswold T. L. (2011). Patterns of widespread decline in North American bumble bees. Proceedings of the National Academy of Sciences.

[B4736425] Campbell J. W., O’Brien J., Irvin J. H., Kimmel C. B., Daniels J. C., Ellis J. D. (2017). Managed bumble bees (*Bombus
impatiens*) (Hymenoptera: Apidae) caged with blueberry bushes at high density did not increase fruit set or fruit weight compared to open pollination. Environmental Entomology.

[B4720619] Agency Canadian Food Inspection Bumblebee Sector Guide To The National Bee Farm-level Biosecurity Standard. http://www.inspection.gc.ca/animals/terrestrial-animals/biosecurity/standards-and-principles/bumblebee-sector-guide/eng/1378396751545/1378397236948?chap=0.

[B4380220] Chandler L. (1956). Parallel color variation in *Bombus
impatiens* Cr. and *Bombus
bimaculatus* (Hymenoptera, Apidae). Proceedings of the Indiana Academy of Science.

[B4720483] Chivian E., Bernstein A. (2008). Sustaining Life: How Human Health Depends on Biodiversity.

[B4702026] Colla Sheila R., Gadallah Fawziah, Richardson Leif, Wagner David, Gall Lawrence (2012). Assessing declines of North American bumble bees (*Bombus* spp.) using museum specimens. Biodiversity and Conservation.

[B4380230] Colla S. R., Otterstatter M. C., Gegear R. J., Thomson J. D. (2006). Plight of the bumble bee: pathogen spillover from commercial to wild populations. Biological Conservation.

[B4736262] Colla S. R., Packer L. (2008). Evidence for decline in eastern North American bumblebees (Hymenoptera: Apidae), with special focus on *Bombus
affinis* Cresson. Biodiversity and Conservation.

[B4720217] Colla S. R., Ratti C. M. (2010). Evidence for the decline of the western bumble bee (*Bombus
occidentalis* Greene) in British Columbia. The Pan-Pacific Entomologist.

[B4736223] COSEWIC (2010). COSEWIC assessment and status report on the Rusty-patched Bumble Bee *Bombus
affinis* in Canada.

[B4736214] COSEWIC (2014). COSEWIC assessment and status report on the Western Bumble Bee *Bombus
occidentalis*, *occidentalis* subspecies (*Bombus
occidentalis
occidentalis*) and the *mckayi* subspecies (*Bombus
occidentalis
mckayi*) in Canada.

[B4736232] COSEWIC (2014). COSEWIC assessment and status report on the Gypsy Cuckoo Bumble *Bombus
bohemicus* in Canada.

[B4736205] COSEWIC (2015). COSEWIC assessment and status report on the Yellow-banded Bumble Bee *Bombus
terricola* in Canada.

[B4379927] Cresson E. T. (1863). List of the North American species of *Bombus* and *Apathus*. Proceedings of the Entomological Society of Philadelphia.

[B4736576] Evison S. E. F., Roberts K. E., Laurenson L., Pietravalle S., Hui J., Biesmeijer J. C., Smith J. E., Budge G., Hughes W. O. H. (2012). Pervasiveness of parasites in pollinators. PLoS One.

[B4379917] Frier S. D., Somers C. M., Sheffield C. S. (2016). Floral longevity, nectar production, anther dehiscence, and stigma receptivity in Haskap (*Lonicera
caerulea*). Journal of Pollination Ecology.

[B4720148] Frier S. D., Somers C. M., Sheffield C. S. (2016). Comparing the performance of native and managed pollinators of Haskap (*Lonicera
caerulea*: Caprifoliaceae), an emerging fruit crop. Agriculture, Ecosystems & Environment.

[B4720492] Gibbs J., Sheffield C. S. (2009). Rapid range expansion of the Wool-Carder Bee, *Anthidium
manicatum* (Linnaeus) (Hymenoptera: Megachilidae), in North America. Journal of the Kansas Entomological Society.

[B4720100] Gibbs J., Dathe H. H. (2017). First records of Hylaeus (Paraprosopis) pictipes Nylander, 1852 (Hymenoptera: Colletidae) in North America. Check List.

[B4720502] Giles V., Ascher J. S. (2006). A survey of the bees of the Black Rock Forest Preserve, New York (Hymenoptera: Apoidea). Journal of Hymenoptera Research.

[B4699238] Goulson D., Hanley M. E., Darvill B., Ellis J. S., Knight M. E. (2005). Causes of rarity in bumblebees. Biological Conservation.

[B4736447] Goulson D. (2010). Impacts of non-native bumblebees in Western Europe and North America. Applied Entomology and Zoology.

[B4380274] Goulson D., Hughes W. O. H. (2015). Mitigating the anthropogenic spread of bee parasites to protect wild pollinators. Biological Conservation.

[B4736285] Graystock P., Yates K., Evison S. E. F., Darvill B., Goulson D., Hughes W. O. H. (2013). The Trojan hives: pollinator pathogens, imported and distributed in bumblebee colonies. Journal of Applied Ecology.

[B4736535] Graystock P., Goulson D, Hughes W. O. H. (2014). The relationship between managed bees and the prevalence of parasites in bumblebees. PeerJ.

[B4736514] Graystock P., Goulson D., Hughes W. O. H. (2015). Parasites in bloom: flowers aid dispersal and transmission of pollinator parasites within and between bee species. Proceedings of the Royal Society B: Biological Sciences.

[B4380250] Graystock P., Blane E. J., McFrederick Q. S., Goulson D., Hughes W. O. H. (2016). Do managed bees drive parasite spread and emergence in wild bees?. International Journal for Parasitology: Parasites and Wildlife.

[B4380284] Grixti J. C., Wong L. T., Cameron S. A., Favret C. (2009). Decline of bumble bees (*Bombus*) in the North American Midwest. Biological Conservation.

[B4700146] Gurevitch J., Padilla D (2004). Are invasive species a major cause of extinctions?. Trends in Ecology & Evolution.

[B4379880] Hicks B. J. (2011). Pollination of lowbush blueberry (*Vaccinium
angustifolium*) in Newfoundland by native and introduced bees. Journal of the Acadian Entomological Society.

[B4379890] Hicks B. J., Sircom J. (2016). Pollination of commercial cranberry (*Vaccinium
macrocarpon* Ait.) by native and introduced managed bees in Newfoundland. Journal of the Acadian Entomological Society.

[B4696509] Hicks B. J., Pilgrim B. L., Perry E., Marshall H. D. (2018). Observations of native bumble bees inside of commercial colonies of *Bombus
impatiens* (Hymenoptera: Apidae) and the potential for pathogen spillover. The Canadian Entomologist.

[B4517845] Hines H. M. (2008). Historical biogeography, divergence times, and diversification patterns of bumble bees (Hymenoptera: Apidae: *Bombus*). Systematic Biology.

[B4720512] Horn T. (2005). Bees in America. How the honey bee shaped a nation.

[B4736297] Ings T. C., Ward N. L., Chittka L. (2006). Can commercially imported bumble bees out-compete their native conspecifics?. Journal of Applied Ecology.

[B4720167] Javorek S. K., Mackenzie K. E., Vander Kloet S. P. (2002). Comparative pollination effectiveness among bees (Hymenoptera: Apoidea) on lowbush blueberry (Ericaceae: *Vaccinium
angustifolium*). Annals of the Entomological Society of America.

[B4736556] Jokela J., Taskinen J., Mutikainen P., Kopp K. (2005). Virulence of parasites in hosts under environmental stress: experiments with anoxia and starvation. Oikos.

[B4736545] Kelly D. W., Paterson R. A., Townsend C. R., Poulin R., Tompkins D. M. (2009). Parasite spillback: a neglected concept in invasion ecology?. Ecology.

[B4517817] Kent C. F., Dey A., Patel H., Tsvetkov N., Tiwari T., MacPhail V. J., Gobeil Y., Harpur B. A., Gurtowski J., Schatz M. C., Colla S. R., Zayed A. (2018). Conservation genomics of the declining North American bumblebee *Bombus
terricola* reveals inbreeding and selection on immune genes. Frontiers in Genetics.

[B4700201] Laate E. A. (2013). The economics of production and marketing of greenhouse crops in Alberta.

[B4379671] Laverty T. M., Harder L. D. (1988). The bumble bees of eastern Canada. The Canadian Entomologist.

[B4720110] Martins K. T., Normandin É., Ascher J. S. (2017). *Hylaeus
communis* (Hymenoptera: Colletidae), a new exotic bee for North America with generalist foraging and habitat preferences. The Canadian Entomologist.

[B4385242] Mitchell T. B. (1962). Bees of the eastern United States. Volume II. North Carolina Agricultural Experiment Station Technical Bulletin.

[B4720129] Normandin É., Vereecken N. J., Buddle C. M., Fournier V. (2017). Taxonomic and functional trait diversity of wild bees in different urban settings. PeerJ.

[B4380294] Otterstatter M. C., Thomson J. D. (2008). Does pathogen spillover from commercially reared bumble bees threaten wild pollinators?. PLoS One.

[B4700166] Pimentel D., Zuniga R., Morrison D. (2005). Update on the environmental and economic costs associated with alien-invasive species in the United States. Ecological Economics.

[B4736591] Power A. G., Mitchell C. E. (2004). Pathogen spillover in disease Epidemics. The American Naturalist.

[B4379816] Ratti C. M., Colla S. R. (2010). Discussion of the presence of an eastern bumble bee species (*Bombus
impatiens* Cresson) in western Canada. The Pan-Pacific Entomologist.

[B4699307] Ricketts T. H., Regetz James, Steffan-Dewenter Ingolf, Cunningham S. A., Kremen Claire, Bogdanski Anne, Gemmill-Herren Barbara, Greenleaf S. S., Klein A. M., Mayfield M. M., Morandin L. A., Ochieng’ Alfred, Viana B. F. (2008). Landscape effects on crop pollination services: are there general patterns?. Ecology Letters.

[B4380304] Sachman-Ruiz B., Narváez-Padilla V., Reynaud E. (2015). Commercial *Bombus
impatiens* as reservoirs of emerging infectious diseases in central México. Biological Invasions.

[B4379745] Sheffield C. S., Kevan P. G., Smith R. F., Rigby S. M., Rogers R. E. L. (2003). Bee species of Nova Scotia, Canada, with new records and notes on bionomics and floralrelations (Hymenoptera: Apoidea). Journal of the Kansas Entomological Society.

[B4720197] Sheffield C. S. (2008). Summer bees for spring crops? Potential problems with *Megachile
rotundata* (Fab.) (Hymenoptera: Megachilidae) as a pollinator of lowbush blueberry (Ericaceae). Journal of the Kansas Entomological Society.

[B4720187] Sheffield C. S., Westby S. M., Kevan P. G., Smith R. F. (2008). Winter management options for the orchard pollinator *Osmia
lignaria* Say (Hymenoptera: Megachilidae) in Nova Scotia. Journal of the Entomological Society of Ontario.

[B4720177] Sheffield C. S., Westby S. M., Smith R. F., Kevan P. G. (2008). Potential of bigleaf lupine for building and sustaining *Osmia
lignaria* populations for pollination of apple. The Canadian Entomologist.

[B4700176] Sheffield C. S., Dumesh S., Cheryomina M. (2011). *Hylaeus
punctatus* (Hymenoptera: Colletidae), a bee species new to Canada, with notes on other non-native species. Journal of the Entomological Society of Ontario.

[B4720207] Sheffield C. S. (2014). Pollination, seed set and fruit quality in apple: studies with *Osmia
lignaria* (Hymenoptera: Megachilidae) in the Annapolis Valley, Nova Scotia, Canada. Journal of Pollination Ecology.

[B4720607] Sheffield C. S., Richardson L., Cannings S., Ngo H., Heron J., Williams P. H. (2016). Biogeography and designatable units of *Bombus
occidentalis* Greene and *B.
terricola* Kirby (Hymenoptera: Apidae) with implications for conservation status assessments. Journal of Insect Conservation.

[B4700156] Shine R. (2010). The ecological impact of invasive cane toads (*Bufo Marinus*) in Australia. The Quarterly Review of Biology.

[B4720085] Sipes J. D., Boylston J. W., Carlton J. T., Fordham M. J., Parsons M. G., Skelton R., Taylor A. H., Thomas E. D., Waite T. D., Weis J. S. (1996). Stemming the Tide. Controlling Introductions of Nonindigenous Speciesby Ships' Ballast Water.

[B4736504] Stephen W. P., Rao S. (2007). Sampling native bees in proximity to a highly competitive food resource (Hymenoptera: Apiformes). Journal of the Kansas Entomological Society.

[B4736437] Strange J. P. (2015). *Bombus
huntii*, *Bombus
impatiens*, and *Bombus
vosnesenskii* (Hymenoptera: Apidae) pollinate greenhouse-grown tomatoes in western North America. Journal of Economic Entomology.

[B4736484] Stubbs C. S., Drummond F. A. (2001). *Bombus
impatiens* (Hymenoptera: Apidae): an alternative to *Apis
mellifera* (Hymenoptera: Apidae) for lowbush blueberry pollination. Journal of Economic Entomology.

[B4736524] Szabo N. D., Colla S. R., Wagner D. L., Gall L. F., Kerr J. T. (2012). Do pathogen spillover, pesticide use, or habitat loss explain recent North American bumblebee declines?. Conservation Letters.

[B4736411] Thorp R. W., Strickler K., Cane J. H. (2003). Bumble bees (Hymenoptera: Apidae): commercial use and environmental concerns. For Nonnative Crops, Whence Pollinators of the Future?.

[B4702014] Velthuis H. H. W., van Doorn A. (2006). A century of advances in bumblebee domestication and the economic and environmental aspects of its commercialization for pollination. Apidologie.

[B4379681] Whidden T. L. (1996). The fidelity of commercially reared colonies of *Bombus
impatiens* Cresson (Hymenoptera: Apidae) to lowbush blueberry in southern New Brunswick. The Canadian Entomologist.

[B4379851] Williams P., Thorp R., Richardson L., Colla S. (2014). Bumble Bees of North America.

[B4720521] Wilson E. O. (1999). The Diversity of Life. New edition.

[B4702004] Work Timothy T., McCullough Deborah G., Cavey Joseph F., Komsa Ronald (2005). Arrival rate of nonindigenous insect species into the United States through foreign trade. Biological Invasions.

